# Nursing Complexity and Health Literacy as Determinants of Patient Outcomes: A Prospective One-Year Multicenter Cohort Study

**DOI:** 10.3390/nursrep15040135

**Published:** 2025-04-17

**Authors:** Antonello Cocchieri, Elena Cristofori, Mario Cesare Nurchis, Nursing and Public Health Group, Gianfranco Damiani, Manuele Cesare

**Affiliations:** 1 Section of Hygiene, Woman and Child Health and Public Health, Gemelli IRCCS University Hospital Foundation, 00168 Rome, Italy; gianfranco.damiani@policlinicogemelli.it (G.D.); manuele.cesare@policlinicogemelli.it (M.C.); 2 Section of Hygiene, Department of Health Science and Public Health, Catholic University of the Sacred Heart, 00168 Rome, Italy; m.nurchis@unilink.it (M.C.N.);; 3 Faculty of Medicine and Surgery, Catholic University of the Sacred Heart, 00168 Rome, Italy; elena.cristofori@unicatt.it; 4 Department of Life Science, Health and Health Professions, Link Campus University, 00165 Rome, Italy

**Keywords:** nursing complexity, nursing diagnosis, health literacy, patient outcome assessment, survival, patient re-admission, emergency room visits

## Abstract

**Background/Objectives:** Although nursing complexity and health literacy (HL) are critical determinants of patient outcomes, their combined impact on mortality, hospital re-admissions, and emergency department (ED) visits remains poorly understood. This study aims to measure nursing complexity and HL in hospitalized patients, examine their interaction, and analyze their impacts on mortality, hospital re-admissions, and ED visits over a one-year follow-up period. **Methods:** Adult patients from two hospital centers were enrolled, excluding those with stays under two days or cognitive impairments. Data were collected at baseline to assess nursing complexity (measured according to the number of nursing diagnoses assigned to patients within 24 h from hospital admission) and HL (assessed using the Single-Item Literacy Screener, SILS). Patients were followed during a 12-month follow-up period to track mortality, hospital re-admissions, and ED visits. Latent class analysis classified patients into distinct nursing complexity and HL profiles. Survival analyses and Cox proportional hazard models were used to evaluate the relationships between variables. **Results:** At baseline, among the 2667 enrolled patients, 55.9% were classified as having high nursing complexity, and 32% had inadequate HL. High nursing complexity was associated with lower HL (r = 0.384; *p* < 0.001). During follow-up, 387 patients (14.5%) were lost. Of the remaining sample, mortality occurred in 8.3% of the patients, hospital re-admissions in 27.2%, and ED visits in 16.8%. Nursing complexity was significantly associated with higher mortality (HR: 1.84, adjusted HR: 1.81), but not with hospital re-admissions or ED visits. The patients with inadequate HL (32%) had increased risks of mortality (HR: 11.21, adjusted HR: 7.75), hospital re-admissions (HR: 3.61, adjusted HR: 3.58), and ED visits (HR: 20.78, adjusted HR: 14.45). The patients with both high nursing complexity and inadequate HL had the highest mortality risk and the lowest 12-month survival rate (75%; 95% CI: 71.1–79.1%; *p* < 0.001). **Conclusions:** This study demonstrates that both high nursing complexity and inadequate HL independently and jointly contribute to adverse patient outcomes. Interventions targeting HL and supporting patients with high nursing complexity could reduce risks, enhance care, and improve patient survival. While these findings underscore the critical role of both factors in patient outcomes, the limitations include this study’s single-country setting and reliance on a single-item HL measure. Future research should validate these findings in broader healthcare contexts and integrate multidimensional HL assessments for a more comprehensive evaluation.

## 1. Introduction

Effective healthcare delivery relies on a complex interplay of factors, among which nursing complexity and health literacy (HL) are pivotal, particularly in hospital settings [1]. Nursing complexity refers to the intricate and multifaceted nature of nursing practice, encompassing numerous interacting components that affect healthcare delivery and patient outcomes [2]. Nursing complexity is recognized as a fundamental attribute of nursing, and several methods have been developed to measure it over time. Although a definitive definition remains elusive, nursing complexity is often assessed through the number of nursing diagnoses (NDs) assigned to each patient and the number of nursing actions (NAs) performed in clinical practice to address NDs [3,4]. Patients with multiple comorbidities and complex clinical conditions typically exhibit higher nursing complexity which, in turn, is associated with an increased risk of adverse health outcomes [3,4,5]. In this regard, research has indicated that patients with high nursing complexity present an increased risk of mortality, prolonged stays, and greater healthcare utilization in hospital settings [4,5,6].

While nursing complexity can reflect the intensity of care required due to underlying clinical conditions, HL directly shapes patient outcomes by affecting their ability to understand and manage health information, adhere to treatment plans, and engage effectively with healthcare systems [7,8,9,10]. Defined as “personal knowledge and competencies obtained through daily activities, social interactions, and across generations”, HL influences an individual’s capacity to navigate healthcare systems, follow treatment plans, and engage in health-promoting behaviors [11,12,13]. In simpler terms, HL refers to a person’s capacity to understand and use health information to make informed decisions about their health [14,15]. Patients with adequate HL are generally better equipped to understand medical conditions and manage their care. This capability can potentially mitigate some of the negative effects of high nursing complexity [16,17]. In contrast, patients with inadequate HL may struggle to comprehend medical information, adhere to treatment recommendations, and make informed decisions [1,11,12], which can exacerbate the challenges associated with nursing complexity. Moreover, inadequate HL is independently linked to increased re-admission rates and mortality in patients with chronic and complex conditions, such as heart failure, chronic obstructive pulmonary disease, diabetes, and cancer, as well as among elderly populations [10,18,19,20].

Considering these premises, addressing the challenges arising from nursing complexity and HL is an urgent priority for healthcare systems. Effectively managing these factors and mitigating their impacts are essential to ensure high-quality care, improve patient safety, and reduce unnecessary healthcare utilization and the overall burden on health systems, both locally and globally [1,7]. However, despite being well-established determinants of patient outcomes, nursing complexity and HL have never been studied together. Most research has analyzed them independently [3,6,9], overlooking their potential combined influence on hospital outcomes, such as mortality, hospital re-admissions, and emergency department (ED) visits, leaving a critical gap in the literature. Considering their distinct yet complementary negative effects on patient outcomes, it is plausible that these factors may interact and jointly influence patient health trajectories. This study addresses this gap by examining how different profiles of nursing complexity and HL interact and contribute to patient outcomes over a one-year follow-up period in a multicenter cohort.

The specific objectives of this study are to (1) describe the nursing complexity and HL levels in hospitalized patients; (2) explore the association between nursing complexity, HL, and patient outcomes, including mortality, hospital re-admissions, and ED visits; and (3) assess the joint impact of nursing complexity and HL on these outcomes over a one-year follow-up period.

## 2. Materials and Methods

### 2.1. Study Design and Setting

This observational prospective cohort study was conducted at two hospital centers in Italy: a large university hospital (with 1611 beds) and a smaller hospital (with 255 beds), both located in Rome. The two hospitals were selected to capture different patient populations, with the university hospital managing complex cases and the smaller hospital reflecting general care, thus enhancing this study’s generalizability. This study comprised two phases: baseline and one-year follow-up data collection. It adhered to the Strengthening the Reporting of Observational Studies in Epidemiology (STROBE) guidelines [21]. The patients were monitored for one year to gather data on mortality, hospital re-admissions, and ED visits.

### 2.2. Study Populations and Recruitment

A consecutive sample of adult patients hospitalized at the two centers was invited to participate. Trained research nurses assessed patient eligibility and informed those who met the inclusion criteria about this study. After obtaining written informed consent, the patients were enrolled within the first 24 h of their hospital admission.

### 2.3. Inclusion Criteria

Patients were included if they were adults (≥18 years old), with a length of stay of at least two days, without documented cognitive impairment, and were able to provide informed consent [1,3].

### 2.4. Exclusion Criteria

The exclusion criteria included patients who died before discharge, experienced disease progression, or withdrew due to caregiver concerns or other personal reasons.

### 2.5. Data Sources and Collection

#### 2.5.1. Baseline Data

**Figure 1:**
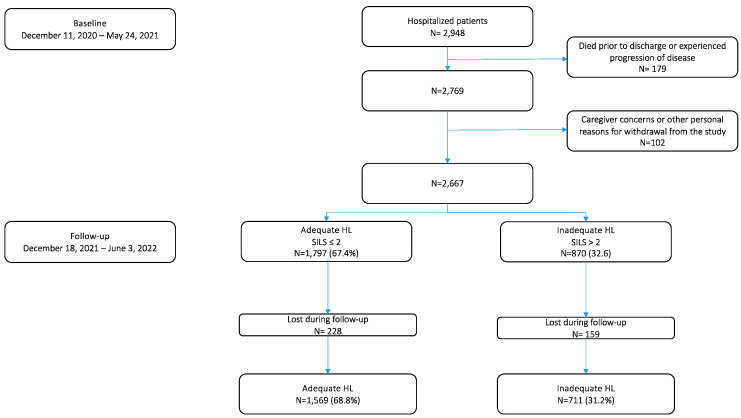
The study’s selection process.

Data were collected at baseline from December 2020 to May 2021 and followed up for one year. A total of 2948 patients initially consented to participate in this study. During the baseline phase, 117 patients died before discharge, and 164 withdrew from this study after initially consenting. Ultimately, 2667 patients were included in this study. The selection process detailing the follow-up procedure is illustrated in Figure 1. Sociodemographic and clinical characteristics were regularly documented in the electronic health records (EHRs). All data were collected in person by trained nurse research assistants.

#### 2.5.2. Follow-Up Data

One-year follow-up data were collected through structured phone interviews from December 2021 to June 2022, ensuring that each participant was contacted one year after being discharged from the hospital. A pre-defined follow-up schedule was implemented, with the patients contacted based on their discharge date (see Supplementary File S1). Ten trained research nurses, following a standardized strategy, verified the discharge dates before conducting interviews to assess patient outcomes, including mortality, hospital re-admissions, and ED visits.

To ensure consistency and accuracy, the nurses underwent a four-hour training program covering this study’s aims, standardized interview techniques, data entry procedures, and the ethical considerations involved in patient follow-up. Training sessions were led by senior researchers to ensure uniformity in data collection and minimize inter-rater variability. Additionally, pilot testing was conducted on a small sample to refine data collection processes before full-scale implementation.

The patients or their caregivers were contacted at scheduled intervals, with up to three attempts made to reach them. If a patient was unreachable, an alternative contact (family member or caregiver) provided at baseline was used. The participants who could not be reached after three attempts within a three-month period were considered lost to follow-up and excluded from further analysis. For the patients who passed away during the study period, mortality was confirmed by family members, following ethical guidelines to maintain sensitivity and confidentiality.

### 2.6. Variables and Measurements

To gather data for this study, the following variables were collected and analyzed:

▪ Nursing complexity, which was assessed by evaluating the number of NDs identified within 24 h of hospital admission and the number of NAs performed throughout the patient’s stay [1,3]. While NDs are standardized clinical judgments that identify patient responses to health conditions and guide individualized care planning, NAs encompass specific tasks and interventions documented in nursing records and performed by nurses as part of their professional responsibilities [3]. These actions are grounded in scientific knowledge, clinical judgment, and a holistic approach to addressing patients’ physical, emotional, social, and spiritual needs [22]. A higher nursing complexity reflects the intensity of required nursing care in hospital settings [4]. This measure has been validated in adult and pediatric populations, demonstrating its reliability in capturing variations in patient care needs across different clinical conditions [3]. Additionally, previous studies have shown that nursing complexity is a significant predictor of key hospital outcomes, including length of stay (LOS) and mortality risk [4,5]. NDs were collected using the Professional Assessment Instrument (PAI), a clinical nursing information system integrated into the hospital’s EHR and used by nurses during their routine practice. The PAI standardizes the nursing diagnostic process by guiding nurses in selecting appropriate NDs based on the assessment data. These suggestions, which can be accepted or rejected by nurses based on their clinical judgment, are provided by a validated algorithm embedded in the PAI system, which has been in use at the study hospitals for over a decade [23]. The PAI system has supported the development of multiple studies since its initial implementation, contributing to research on NDs, nursing complexity, and patient outcomes [1,3,4].
▪ HL, which was collected during hospitalization using the Single-Item Literacy Screener (SILS) as the assessment tool [24]. The SILS is commonly used in clinical settings to evaluate an individual’s understanding of health information. It consists of a single question: “How often do you ask someone for help to read the instructions and leaflets from a doctor or pharmacy?” Responses are given on a 5-point Likert scale, ranging from “never” to “always”. A response of “never” (1) or “rarely” [25] indicates adequate HL, while responses of “sometimes” (3), “often” (4), and “always” (5) suggest potential difficulties with reading health-related materials. Scores above 2 on the SILS are used to identify patients with low HL. The Italian version of the SILS, developed and validated in 2017, demonstrates good concurrent validity when compared with the Newest Vital Sign (r = −0.679; *p* < 0.001) and shows high diagnostic accuracy, with a sensitivity of 83.3% and a specificity of 82.6% within the general population [26]. The SILS is considered an efficient method for assessing HL, offering a straightforward alternative to more comprehensive instruments that measure functional HL.

### 2.7. Outcomes Measured

Study outcome data (mortality, hospital re-admissions, and ED visits) were collected throughout the follow-up period. The primary outcome was mortality, defined as death from any cause and measured from hospital discharge to death in months. Secondary outcomes included the time to the first re-admission (any hospitalization) and time to the first ED visit (any access to the ED), both occurring during the follow-up period.

### 2.8. Covariate Variables

Covariates were selected based on their anticipated relationships with nursing complexity, HL, and the study outcomes. The following variables were documented during hospitalization and extracted from the EHRs:

▪ Sociodemographic characteristics. These included age, gender, education level, monthly family income, and place of origin (rural–urban classification).
▪ Clinical characteristics. This category included the modality of hospital admission (planned or emergency through ED), the number of chronic conditions, and LOS.
▪ Major diagnostic categories (MDCs). MDCs categorize ICD-9-CM medical diagnoses into 25 groups. Each MDC aligns with a specific medical specialty and is associated with a particular organ system or etiology. Diagnoses within an MDC share common characteristics related to the affected organ system or underlying cause, distinguishing them from diagnoses in other MDCs.

### 2.9. Statistical Analysis

The Kolmogorov–Smirnov test was used to assess the normality of the distributions of the quantitative variables. The continuous variables that were found to be normally distributed, such as the SILS score, NDs, and NAs, are described using means and standard deviations (SDs). Meanwhile, the median and interquartile range (IQR) were calculated for the variables that were not normally distributed, such as age and LOS. The categorical variables, including gender, education, MDCs, and type of admission, are described using counts and percentages (specific objective 1). The association between the continuous and categorical variables was assessed using parametric tests (Student’s *t*-test and ANOVA) for normally distributed data and or non-parametric tests (Mann–Whitney or Kruskal–Wallis) for non-normally distributed variables. Student’s *t*-test was used to compare ND and NA scores between the adequate and low HL groups. Non-parametric tests, such as chi-squared or Fisher’s tests, were used to compare differences in the number of chronic conditions, admission modality, and MDCs between the adequate HL and low groups. The Mann–Whitney U-test was used to compare two independent samples (adequate HL group vs. low HL group), while the Kruskal–Wallis H-test was used to analyze LOS and age across different HL scores and categories. Pearson’s correlation analysis was performed to explore the relationship between NDs and HL (specific objective 2). LCA was used to identify distinct profiles based on the number of NDs and NAs [3]. Multiple models with varying numbers of classes (from 2 to 4) were estimated. Model fit was assessed using the Akaike information criterion (AIC), the Bayesian information criterion [27], and entropy. A lower AIC/BIC indicates a better model fit, and a higher entropy suggests clearer class separation. The analysis indicated that a 2-class solution best fit the data, with an entropy value of 0.945, indicating good class separation. LCA was used to categorize the patients into low- and high-complexity groups based on patterns in the observed data; in particular, the patients were categorized into “low nursing complexity” (low number of NDs and NAs) and “high nursing complexity” (higher number of NDs and NAs). The patients were further stratified into four groups based on nursing complexity and HL levels: (A) low nursing complexity with adequate HL, (B) low nursing complexity with inadequate HL, (C) high nursing complexity with adequate HL, and (D) high nursing complexity with inadequate HL. Kaplan–Meier plots and log-rank tests were used to illustrate and assess group survival differences. Cox proportional hazards regression models were employed to analyze time-to-event survival data (specific objective 3), both in unadjusted models and in models adjusted for key covariates, including age, gender, education level, monthly family income, rural–urban classification, type of hospital admission, number of chronic conditions, and length of stay. These covariates were selected based on their theoretical and empirical relevance to nursing complexity, HL, and patient outcomes [3,7,28]. The data analysis was conducted using Jamovi (version 2.4). All tests were two-sided. A *p*-value less than 0.05 was considered statistically significant.

### 2.10. Ethical Considerations

Ethical approval for this study was granted by the Catholic University of the Sacred Heart Ethics Committee (Protocol No. 0015416/21). Prior to any data collection, all the participants provided written informed consent, ensuring that they were fully aware of this study’s objectives and procedures and their rights, including the right to withdraw from this study at any time without any negative consequences. This study is follow-up research, and the participants were informed, during the initial phase, about the potential for future contact regarding continued research, for which they provided explicit consent. Data collected during the follow-up phase were securely stored in a password-protected database compliant with data protection regulations, with access limited to authorized research personnel. The participants who could not be reached after three contact attempts within a three-month period were excluded from this study. To safeguard privacy, all personal identifiers were removed, and data were anonymized throughout the research process. This study adhered to the principles of the Declaration of Helsinki and good clinical practice, with a strong focus on maintaining the participants’ confidentiality and safeguarding their well-being throughout this research.

## 3. Results

### 3.1. General Characteristics of the Sample

At baseline, a total of 2948 patients were initially approached for participation. Of these, 179 died before discharge and 102 withdrew after initial consent, resulting in a final study sample of 2667 patients. The sample was predominantly female (54.1%), with a median age of 65 years (IQR, 23 years). The most common educational level was less than a high school diploma (43.1%). More than half of the participants (55.4%) reported a middle-income range per month (EUR 1001–2000), and a significant portion lived in urban areas (48.1%). Scheduled admissions accounted for 80% of the patient entries, and more than half (58.5%) had at least one chronic illness. The most frequent causes of hospitalization were hepatobiliary and pancreatic diseases and disorders (DDs), cardiocirculatory system DDs, and respiratory system MDCs, representing more than 10% of the cases (Table 1).

### 3.2. Nursing Complexity and HL Levels of the Sample

The mean number of NDs in the overall sample was 4.12 (SD: 3.0). LCA categorized the patients into two groups: “low nursing complexity” and “high nursing complexity” (AIC = 4202; BIC = 4608; entropy = 0.945, indicating a good classification solution). Of the sample, 1491 (55.9%) patients were classified as high nursing complexity, while 1176 (44.1%) were classified as low nursing complexity. No significant demographic or clinical differences were found between the low and high nursing complexity groups.

The mean score on the SILS was 1.97 (SD: 1.21). Approximately 32% of the patients had low HL (SILS > 2), with 870 patients (32.6%) falling into this category. Statistically significant differences were observed between the patients with adequate and inadequate HL in most sociodemographic and clinical characteristics, except for monthly family income (*p* = 0.949) and patient provenance (*p* = 0.309); see Table 1. An analysis of covariates showed that older patients and those with lower education levels were more likely to have both inadequate HL and high nursing complexity. Additionally, 58% of the patients had at least one chronic illness, with a higher prevalence among those with inadequate HL (see Table 1).

In the bivariate analysis, a higher number of NDs was associated with a higher SILS score, indicating that the patients with high nursing complexity generally had worse HL (Pearson’s correlation coefficient r = 0.384; *p* < 0.001). The patients were categorized into four groups based on nursing complexity and HL levels: 828 patients (31.0%) were classified as low nursing complexity with adequate HL, 348 (13.0%) as low nursing complexity with inadequate HL, 969 (36.3%) as high nursing complexity with adequate HL, and 522 (19.6%) as high nursing complexity with inadequate HL. Comparisons among these four groups revealed significant differences in all the studied variables, except for family income and patient provenance (Table 2).

### 3.3. Nursing Complexity, Mortality, Hospital Re-Admissions, and ED Visits

During the follow-up period, 387 participants (14.5%) were lost to follow-up. Of the remaining sample, 189 patients (8.3%) died from any cause, 621 individuals (27.2%) were re-admitted, and 333 participants (16.8%) visited the ED. Mortality rates differed significantly between the patients with low and high nursing complexity (5.7% vs. 10.3%, respectively; *p* < 0.001). However, there were no significant differences in hospital re-admissions or ED visits between the two groups (26.7% vs. 27.6%, respectively; *p* = 0.629). In a Cox proportional hazard model, the patients with high nursing complexity had a 1.84-fold increased risk of mortality compared to those with low nursing complexity. After adjusting for covariates, the mortality risk for the patients with high nursing complexity was 1.81 times greater than for those with low nursing complexity (Table 3).

### 3.4. HL, Mortality, Hospital Re-Admissions, and ED Visits

During the follow-up period, significant differences were observed between the patients with inadequate HL and those with adequate HL in terms of mortality (21.9% vs. 2.1%; *p* < 0.001), hospital re-admissions (50.2% vs. 16.8%; *p* < 0.001), and ED visits (40.5% vs. 3.5%; *p* < 0.001); see Table 4.

In a simple Cox regression model, the patients with inadequate HL had an 11.21-fold increased risk of mortality compared with those with adequate HL. After adjusting for covariates, the risk was reduced to 7.75-fold. For hospital re-admissions and ED visits, the patients with inadequate HL faced a 3.61-fold higher risk of re-admission and a 20.78-fold higher risk of ED visits. After adjusting for covariates, these risks were moderated to 3.58-fold and 14.45-fold, respectively (Table 5).

### 3.5. Association Between Nursing Complexity, HL, and Mortality

**Figure 2:**
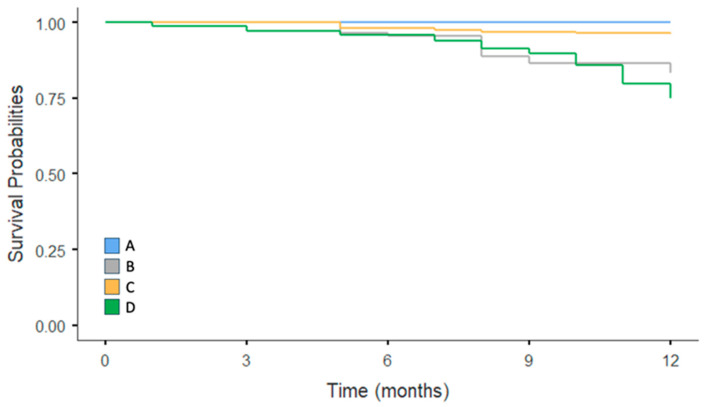
Kaplan–Meier survival curves for mortality by nursing complexity and HL levels. Note: (A) patients with low nursing complexity and adequate HL; (B) patients with low nursing complexity and inadequate HL; (C) patients with high nursing complexity and adequate HL; and (D) patients with high nursing complexity and inadequate HL.

The patients were classified into four groups based on their nursing complexity and HL levels, and Kaplan–Meier survival curves and log-rank tests were used to compare mortality risks across these groups (see the Methods Section). Group D, comprising patients with high nursing complexity and inadequate HL, demonstrated the highest risk of mortality among all the groups (*p* < 0.001); see Figure 2. The 12-month survival rate for this group was 75% (95% CI: 71.1–79.1%; *p* < 0.001).

In summary, this study identified significant associations between nursing complexity, HL, and key patient outcomes. The patients with high nursing complexity had an increased risk of mortality, although no significant differences were observed in hospital re-admissions or ED visits. In contrast, inadequate HL was strongly associated with a higher risk across all three outcomes—mortality, hospital re-admissions, and ED visits. The patients presenting both high nursing complexity and inadequate HL showed the highest risk of mortality.

## 4. Discussion

This study aimed to describe the levels of nursing complexity and HL in hospitalized patients, explore the relationship between these factors and health outcomes, and, finally, evaluate their impacts on mortality, hospital re-admissions, and ED visits. The results provide significant insights into how nursing complexity and HL independently and collectively influence patient outcomes.

According to the literature [3,4], this study identified nursing complexity by analyzing the number of NDs per patient recorded at hospital admission, revealing significant variations in nursing complexity across different inpatient units and medical diagnoses. Although similar methods have been used in previous pediatric studies [3], to the best of our knowledge, this is the first study to apply LCA to classify adult patients based on both the number of NDs recorded at admission and the number of NAs documented throughout the entire hospitalization period, allowing for the identification of patient groups with low and high nursing complexity. Notably, this study revealed that a significant proportion of the patients (55.9%) exhibited high nursing complexity, highlighting the extensive care requirements of a large segment of the hospitalized population and the need for close monitoring and interventions. These results align with a recent study by Ausili et al. [6], who also identified high care needs across various patient groups. While various methods and tools are employed to define and measure nursing complexity, the literature consistently highlights the importance of its assessment for the accurate prediction of patient outcomes.

Approximately one-third of the patients (32.6%) exhibited inadequate HL, with significant sociodemographic and clinical differences compared to those with adequate HL. For instance, the patients with inadequate HL tended to be older, less educated, and had a higher prevalence of chronic conditions, all of which can intensify the challenges in managing their health and navigating the healthcare system. Previous studies on HL in hospitalized patients have reported similar findings, with 29% to 32% of patients showing inadequate HL [1,9]. This prevalence is notably higher than that found in the general population, emphasizing the increased vulnerability of hospitalized patients, who often face more complex healthcare challenges. This underscores the necessity of systematic assessment and targeted interventions to address HL within hospital settings.

This study’s findings regarding the impacts of nursing complexity and HL on mortality, hospital re-admissions, and ED visits are particularly significant. They revealed a strong association between higher nursing complexity and inadequate HL, reinforcing that patients requiring more complex nursing care often face difficulties in understanding and managing their health conditions [1,29,30]. These patients tend to be more frail and are at risk of negative outcomes [1].

The patients with high nursing complexity showed a significantly increased risk of mortality compared with those with lower complexity, even after adjusting for relevant covariates. This finding is consistent with a previous study that reported similar results [6]. Interestingly, despite the strong link to mortality, no significant differences were observed in hospital re-admissions or ED visits, which was an unexpected outcome.

Similarly, the patients with inadequate HL faced significantly higher risks across all measured outcomes—that is, mortality, hospital re-admissions, and ED visits—compared to those with adequate HL. The adjusted Cox regression models indicated that inadequate HL increased mortality risk by 7.75 times, hospital re-admissions by 3.58 times, and ED visits by 14.45 times. These findings are consistent with previous studies that have demonstrated the significant impact of HL on health outcomes [9,11,12,31]. Patients with lower HL may require additional resources, support, and enhanced discharge planning to ensure a smooth transition from hospital to outpatient care. To optimize long-term self-management or care models, the involvement of a multidisciplinary team of professionals to coordinate and plan for sustained care is essential.

This study is the first to combine nursing complexity and HL as criteria for categorizing distinct patient groups and analyzing their associated health outcomes. By integrating these two dimensions, this study provides a novel perspective on the complex challenges faced by different patient populations and their subsequent impacts on health outcomes.

The interaction between nursing complexity and HL may be driven by several inter-related factors. Patients with high nursing complexity often present multiple comorbidities and complex care needs, requiring extensive nursing interventions [4]. For individuals with inadequate HL, navigating these complex care regimens can be particularly challenging, leading to difficulties in following treatment plans, understanding medical instructions, and making informed health decisions [7,32]. Inadequate HL may also limit the patient’s ability to engage in shared decision-making and self-care [33,34], ultimately exacerbating the challenges posed by high nursing complexity.

Our findings confirm the hypothesis that inadequate HL exacerbates the challenges faced by patients with high nursing complexity. Moreover, our results show that patients with both high nursing complexity and inadequate HL are at greater risk of adverse outcomes, including higher mortality rates and a greater likelihood of hospital re-admissions and ED visits. This suggests that these dimensions do not operate in isolation but, rather, interact to amplify risks. Conceptually, inadequate HL may lead to difficulties in understanding medical instructions, following treatment regimens, and recognizing early signs of health deterioration, thereby increasing the burden on nursing care and necessitating more advanced and intensive interventions and education [7,35]. This reinforces the need for an integrated approach to patient care, wherein HL is routinely assessed alongside nursing complexity to identify at-risk populations and implement targeted and multidisciplinary interventions. Additionally, tailored discharge plans and appropriate referrals to other healthcare facilities are essential to address both the clinical and educational needs of vulnerable populations, ultimately improving health outcomes. This research aligns with the public health goals of enhancing patient education and empowerment, reducing healthcare costs, and improving the quality of care.

### 4.1. Study Contributions, Limitations, and Recommendations for Further Research

This study provides a novel perspective by integrating nursing complexity and health literacy (HL) as key determinants of patient outcomes. Through the application of a dual-factor methodology, it offers a deeper understanding of how these factors interact and influence patient survival, hospital re-admission rates, and emergency department (ED) visits. This study’s strengths include its prospective cohort design with a large sample of 2667 patients and a one-year follow-up period, enhancing the reliability of the findings. Additionally, the use of advanced statistical techniques, such as Cox proportional hazards models and latent class analysis (LCA) to classify nursing complexity, strengthens the validity of the results. By focusing on critical patient outcomes, this study has direct implications for the improvement of care strategies, particularly for patients with complex healthcare needs.

However, several limitations should be acknowledged. First, this study was conducted in two hospital centers in Italy, which may limit the generalizability of the findings to healthcare systems with different infrastructures, resource availability, and patient demographics. To strengthen the generalizability of our findings, future research should focus on externally validating this model in diverse healthcare systems and cultural contexts. Multicenter studies conducted in different countries, including those with varied organizational structures and patient populations, would be essential to assess the robustness of our findings. Additionally, cross-national collaborations could explore the influence of healthcare policies, resource availability, and patient engagement strategies on the relationship between nursing complexity, HL, and patient outcomes.

Second, although multiple covariates were adjusted for, unmeasured confounders—such as the patient’s adherence to treatment, social support, and the quality of provider–patient communication—may have influenced the results.

Third, while the Single-Item Literacy Screener (SILS) is a validated and practical tool for assessing HL, it captures only a single dimension of HL, potentially underestimating its broader impact on patient outcomes. Specifically, the SILS primarily assesses reading ability rather than the full spectrum of HL, which includes comprehension, numeracy, and decision-making in healthcare contexts [26]. This distinction is important, as individuals with limited reading skills may still effectively understand and manage their health through verbal communication, while others with adequate literacy may struggle with applying medical and nursing information. Future research should incorporate multidimensional HL assessment tools, such as the Newest Vital Sign (NVS) [1], to provide a more comprehensive evaluation.

Fourth, there is a potential for recall bias, as follow-up interviews were conducted one year after hospitalization. Research suggests that recall accuracy may decline over extended periods, particularly for complex health-related events, potentially affecting the reliability of self-reported outcomes [36]. The patients and caregivers may have had difficulty accurately recalling previous events, which could have influenced the reliability of the obtained data. Additionally, if a patient was unreachable, an alternative family member or caregiver was contacted. While this ensured data completeness, it may have introduced variability in responses, as caregivers may not always accurately recall the patient’s experiences and clinical status [37]. Future research should consider shorter follow-up intervals or alternative data collection methods, such as linking EHRs or integrating national health database linkages, in order to enhance the accuracy of the obtained data.

Fifth, the hazard ratios observed for the association between inadequate HL and ED visits were exceptionally high. While statistically significant and consistent with prior research on HL and health service utilization, such magnitudes are uncommon in research on health outcomes. These results may reflect residual confounding, potential misclassification due to the use of a single-item HL tool, or selection bias. Accordingly, these findings should be interpreted with caution and validated in future studies using multidimensional HL measures.

Finally, the study period coincided with the COVID-19 pandemic, which may have influenced both patient outcomes and the complexity of nursing care [38], thus potentially affecting this study’s findings. Future studies should explore the interplay between global health crisis situations, nursing complexity, and patient outcomes to provide a more complete understanding of these dynamics.

### 4.2. Implications for Policy, Education, and Practice

The findings of this study have important implications for policy and clinical practice, particularly in the management of patients with high nursing complexity and inadequate HL. Integrating HL assessments into routine clinical evaluations could help healthcare providers identify patients at greater risk for poor outcomes, such as higher mortality, hospital re-admissions, and ED visits. Early identification of these at-risk patients would allow clinicians to implement targeted interventions to enhance their HL, such as simplifying medical information, offering additional resources, and ensuring clear and effective communication.

Our results indicate a need for targeted education strategies that enable nurses to assess and manage nursing complexity in a structured and consistent way, ensuring that NDs accurately reflect patient needs. Such training should also prepare nurses to recognize the added challenges posed by inadequate HL [35] and to plan and deliver care that is responsive to both complexity and patients’ capacities to engage in their care. Building on this, training programs should incorporate structured approaches to the early identification of patients with high nursing complexity and low HL, enabling timely and appropriate care adjustments. In addition, continuing education initiatives should enhance healthcare professionals’ communication skills, allowing them to convey complex health information in accessible and patient-centered ways for individuals with varying levels of HL.

Through the integration of HL screening into routine clinical assessments and reinforcing education focused on nursing complexity, healthcare professionals can play a key role in mitigating the negative effects of these factors on patient outcomes. This approach not only enhances patient survival and reduces healthcare utilization, but it also aligns with the broader public health goals of improving patient empowerment and quality-of-care delivery.

## 5. Conclusions

This study underscored the critical influence of both nursing complexity and HL on mortality, hospital re-admissions, and ED visits. The findings demonstrated the importance of addressing both factors in clinical practice to improve patient outcomes. However, this study’s limitations—including its single-country setting and the use of a single-item HL measure—should be considered when interpreting the results. Future research should validate these findings in different healthcare settings and incorporate multidimensional HL assessments. This study suggested strategies to guide future research and clinical interventions. In particular, integrating HL assessments into routine evaluations and tailoring interventions to enhance HL—especially for patients with complex care needs—can lead to better survival rates and optimize healthcare delivery. The results also suggested that improving HL through targeted educational strategies could help patients to better manage their complex care needs, thereby reducing the risk of adverse outcomes associated with high nursing complexity, thus aligning with broader public health goals aimed at enhancing care quality and patient empowerment.

## Figures and Tables

**Table 1:** Sample characteristics and comparisons between adequate and inadequate HL groups.

	General Sample (N = 2667)	Adequate HL (N = 1797)	Inadequate HL (N = 870)	*p*-Value ^a^
**Age**, median (IQR), years	65 (23)	61 (23)	73 (17)	<0.001
**Gender**				
Male	1224 (45.9)	774 (43.1)	450 (51.7)	<0.001
Female	1443 (54.1)	1023 (56.9)	420 (48.3)	
**Education**				<0.001
Less than high school	1149 (43.1)	639 (35.6)	510 (58.6)	
High school	1116 (41.8)	858 (47.7)	258 (29.7)	
University degree	378 (14.2)	282 (15.7)	96 (11.0)	
No education	24 (0.9)	18 (1.0)	6 (0.7)	
**Family income per month** (euros) (N = 2655)				0.949
0–1000	636 (24.0)	425 (23.8)	211 (24.3)	
1001–2000	1472 (55.4)	994 (55.6)	478 (55.1)	
>2000	547 (20.6)	369 (20.6)	178 (20.5)	
**Rural–urban classification**				0.309
City	1281 (48.1)	863 (48.1)	418 (48.1)	
Town	1067 (40.1)	730 (40.7)	337 (38.8)	
Rural area	315 (11.8)	201 (11.2)	114 (13.1)	
**Modality of admission**				<0.001
Planned admission	2148 (80.5)	1512 (84.1)	636 (73.1)	
From ED	519 (19.5)	285 (15.9)	234 (26.9)	
**Number of chronic conditions**				<0.005
0	1105 (41.4)	780 (43.3)	325 (37.4)	
1	412 (15.4)	284 (15.8)	128 (14.7)	
≥2	1150 (43.1)	733 (40.8)	417 (47.9)	
**LOS**, median (IQR), years	4 (6)	4 (5)	6 (9)	<0.001
**MDC**				<0.001
Hepatobiliary and pancreatic DDs	570 (21.4)	300 (16.7)	270 (31.0)	
Cardiocirculatory system DDs	447 (16.8)	381 (21.2)	66 (7.6)	
Respiratory system DDs	315 (11.8)	267 (14.9)	48 (5.5)	
Skin, subcutaneous tissue, and breast DDs	264 (9.9)	180 (10.0)	84 (9.7)	
Nervous system DDs	231 (8.7)	105 (5.8)	126 (14.5)	
Digestive system DDs	198 (7.4)	108 (6.0)	90 (10.3)	
Musculoskeletal and connective system DDs	171 (6.4)	117 (6.5)	54 (6.2)	
Ear, nose, mouth, and throat DDs	153 (5.7)	93 (5.2)	60 (6.9)	
Myeloproliferative DDs, poorly differentiated neoplasms	111 (4.2)	81 (4.5)	30 (3.4)	
Reproductive system DDs	75 (2.8)	63 (3.5)	12 (1.4)	
Infectious and parasitic, systemic, or unspecified site DDs	69 (2.6)	51 (2.8)	18 (2.1)	
Other	63 (2.3)	51 (2.9)	12 (1.4)	
**NDs**, mean (SD)	4.12 (3.0)	3.64 (1.89)	5.11 (4.33)	<0.001
**NAs**, mean (SD)	7.05 (4.32)	6.81 (3.28)	7.55 (5.88)	<0.001

Abbreviations: ED = emergency department; LOS = length of stay; IQR = interquartile range; MDC = major diagnostic category; DDs = diseases and disorders; NDs = nursing diagnoses; SD = standard deviation; NAs = nursing activities; HL = health literacy. ^a^ = U, Mann–Whitney; Fisher’s Exact Test; *t*-student; chi-squared test.

**Table 2:** Comparison of characteristics among the four groups stratified by nursing complexity and HL at baseline.

	General Sample (N = 2667)	Low Nursing Complexity (N = 1176)		High Nursing Complexity (N = 1491)		*p*-Value ^a^
		Adequate HL (N = 828)	Inadequate HL (N = 348)	Adequate HL (N = 969)	Inadequate HL (N = 522)	
**Variables**		A	B	C	D	
**Age**, median (IQR), years	65 (23)	61 (25)	73 (15)	62 (21)	73.5 (20)	
**Gender**						<0.001
Male	1224 (45.9)	375 (45.3)	174 (50.0)	399 (41.2)	276 (52.9)	
Female	1443 (54.1)	453 (54.7)	174 (50)	570 (58.8)	246 (47.1)	
**Education**						
Less than high school	1149 (43.1)	327 (39.5)	186 (53.4)	312 (32.2)	324 (62.1)	
High school	1116 (41.8)	387 (46.7)	111 (31.9)	471 (48.6)	147 (28.2)	
University degree	378 (14.2)	102 (12.3)	51 (14.7)	180 (18.6)	45 (8.6)	
No education	24 (0.9)	12 (1.4)	0 (0)	6 (0.6)	6 (1.1)	<0.001
**Family income per month** (euros) (N = 2655)						0.599
0–1000	636 (24.0)	208 (25.3)	82 (23.6)	217 (22.4)	129 (24.8)	
1001–2000	1472 (55.4)	437 (53.2)	189 (54.5)	557 (57.6)	289 (55.6)	
>2000	547 (20.6)	176 (21.4)	76 (21.9)	193 (20.0)	102 (19.6)	
**Rural–urban classification**						0.426
City	1281 (48.1)	398 (48.1)	172 (49.4)	465 (48.1)	246 (47.2)	
Town	1067 (40.1)	327 (39.5)	135 (38.8)	403 (41.7)	202 (38.8)	
Rural area	315 (11.8)	103 (12.4)	41 (11.8)	98 (10.1)	73 (4.0)	
**Modality of admission**						<0.001
Planned admission	2148 (80.5)	657 (79.3)	273 (78.4)	855 (88.2)	363 (69.5)	
From ED	519 (19.5)	171 (20.7)	75 (21.6)	114 (11.8)	159 (30.5)	
**Number of chronic conditions**						<0.005
0	1105 (41.4)	351 (42.4)	145 (41.7)	429 (44.3)	180 (34.5)	
1	412 (15.4)	139 (16.8)	54 (15.5)	145 (15.0)	74 (14.2)	
≥2	1150 (43.1)	338 (40.8)	149 (42.8)	395 (40.8)	268 (51.3)	
**LOS**, median (IQR), years	4(6)	4 (5)	5 (10)	3 (6)	7 (9)	<0.001
**MDC**						<0.001
Hepatobiliary and pancreatic DDs	570 (21.4)	165(19.9)	165 (47.4)	135 (13.9)	105 (20.1)	
Cardiocirculatory system DDs	447 (16.8)	180 (21.7)	0 (0)	201 (20.7)	66 (12.6)	
Respiratory system DDs	315 (11.8)	75 (9.1)	9 (2.6)	192 (19.8)	39 (7.5)	
Skin, subcutaneous tissue, and breast DDs	264 (9.9)	90 (10.9)	33 (9.5)	90 (9.3)	51 (9.8)	
Nervous system DDs	231 (8.7)	69 (8.3)	57 (16.4)	36 (3.7)	69 (13.2)	
Digestive system DDs	198 (7.4)	39 (4.7)	42 (12.1)	69 (7.1)	48 (9.2)	
Musculoskeletal and connective system DDs	171 (6.4)	54 (6.5)	12 (3.4)	63 (6.5)	42 (8.0)	
Ear, nose, mouth, and throat DDs	153 (5.7)	39 (4.7)	18 (5.2)	54 (5.6)	42 (8.0)	
Myeloproliferative DDs, poorly differentiated neoplasms	111 (4.2)	48 (5.8)	0 (0)	33 (3.4)	30 (5.7)	
Reproductive system DDs	75 (2.8)	42 (5.1)	9 (2.6)	21 (2.2)	3 (0.6)	
Infectious and parasitic, systemic, or unspecified site DDs	69 (2.6)	21 (2.5)	3 (0.9)	30 (3.1)	15 (2.9)	
Other	63 (2.3)	6 (0.8)	0 (0.0)	45 (4.7)	12 (2.4)	
**NAs** (number of activities documented in HR) Mean (SD)	7.05 (4.32)	3.34 (4.13)	4.85 (3.05)	8.49 (2.44)	10.4 (5.15)	<0.001

Abbreviations: HL = health literacy; IQR = interquartile range; LOS = length of stay; ED = emergency department; MDC = major diagnostic category; DDs = diseases and disorders; NAs = nursing activities; HR = health record; SD = standard deviation. ^a^ = Kruskal–Wallis; chi-squared test; ANOVA.

**Table 3:** Cox regression models: NDs on mortality (N = 2280).

Variables	Hazard Ratio	95% CI	*p*-Value
**Simple Cox regression without adjustment**			
**Nursing complexity (NDs) ^a^**			
Low nursing complexity	1.0		
High nursing complexity	1.84	1.35–2.51	<0.001
**Cox Regression with adjustment ^†^**			
**Nursing complexity (NDs) ^b^**			
Low nursing complexity	1.0		
High nursing complexity	1.81	1.32–2.48	<0.001

Abbreviations: NDs = nursing diagnoses; CI = confidence interval. Note: ^†^ = Adjustment for covariates: age, gender, education, family income per month, rural–urban classification, hospital admission, number of chronic conditions, and length of stay. Statistics; ^a^ = $\chi^{2}$ = 15.819; *p* < 0.001; ^b^ = $\chi^{2}$ = 31.797; *p* < 0.001.

**Table 4:** HL levels and study outcomes (N = 2280).

	General Sample (N = 2280)	Adequate HL (N = 1569)	Inadequate HL (N = 711)	*p*-Value ^a^
Mortality (1 year)	189 (8.3)	33 (2.1)	156 (21.9)	<0.001
Re-admission (1 year)	621 (27.2)	264 (16.8)	357 (50.2)	<0.001
ED visits (1 year)	333 (16.8)	45 (3.5)	288 (40.5)	<0.001

Abbreviations: HL = health literacy, ED = emergency department. ^a^ = chi-squared test.

**Table 5:** Cox regression models: HL on study outcomes (N = 2280).

	Mortality			Re-Admissions			ED Visits		
Variables	HR	95% CI	*p*-Value	HR	95% CI	*p*-Value	HR	95% CI	*p*-Value
**Simple Cox regression without adjustment**									
**HL**									
Adequate	1.0			1.0			1.0		
Inadequate	11.21	7.70–16.32	<0.001	3.61	3.06–4.20	<0.001	20.78	14.16–30.50	<0.001
	$\chi^{2}$ = 23.059		<0.001	$\chi^{2}$ = 37.962		<0.001	$\chi^{2}$ = 42.330		<0.001
**Cox regression with adjustment ^†^**									
**HL**									
Adequate	1.0			1.0			1.0		
Inadequate	7.75	5.25–11.45	<0.001	3.58	2.95–4.10	<0.001	14.45	10.52–19.86	<0.001
	$\chi^{2}$ = 36.863		<0.001	$\chi^{2}$ = 51.532		<0.001	$\chi^{2}$ = 66.621		<0.001

Abbreviations: ED = emergency department; HR = hazard ratio; CI = confidence interval. Note: ^†^ = Adjustment for covariates: age, gender, education, family income per month, rural–urban classification, hospital admission, number of chronic conditions, and length of stay.

## Data Availability

The data presented in this study are available on request from the corresponding author due to privacy and ethical reasons.

## Supplementary Materials

The following supporting information can be downloaded at: https://www.mdpi.com/article/10.3390/nursrep15040135/s1, File S1: Follow-Up Data Collection Tool—HL_01.

## Public Involvement Statement

There was no public involvement in any aspect of this research.

## Guidelines and Standards Statement

This manuscript was drafted following the STROBE guidelines [21] for observational research.

## Abbreviations

The following abbreviations are used in this manuscript:

AIC	Akaike information criterion
BIC	Bayesian information criterion
CI	confidence interval
COVID-19	Coronavirus disease 2019
DDs	diseases and disorders
ED	emergency department
EHRs	electronic health records
HL	health literacy
HR	hazard ratio
IQR	interquartile range
LCA	latent class analysis
LOS	length of stay
MDC	major diagnostic category
NDs	nursing diagnoses
SD	standard deviation
SILS	Single-Item Literacy Screener
STROBE	Strengthening the Reporting of Observational Studies in Epidemiology
