# Supplementary material for: Nursing Complexity and Health Literacy as Determinants of Patient Outcomes: A Prospective One-Year Multicenter Cohort Study
Source: Nurs Rep. 2025 Apr 17;15(4):135. doi: 10.3390/nursrep15040135 (PMC12029856; doi:10.3390/nursrep15040135)
Supplement: Supplementary file 1 [file nursrep-15-00135-s001.zip › nursrep-3568785-supplementary.pdf]

# Follow-Up Data Collection Tool – HL_01

| **Patient Name:** | **Follow-Up ID** (same as Database_HL_01): |
|---|---|
| **Date of Phone Follow-Up:** | |
| **Phone Number:** | |

- *Hello, I am calling from ……. regarding a previous hospital admission at …….*
- *May I speak with Mr./Ms.* ______________________________________________?

1 □ Alive

2 □ Deceased

**If the patient is deceased:**

*I am very sorry. Could you tell me the date of death?* Date of death: ____________________

**If the patient is alive:**

*We would like to ask just a few questions about your health over the past year since your hospital discharge……*

- **Emergency Department Visits in the Past 12 Months**

*Did you go to the Emergency Department at any time during the past year?*

1 □ Yes Date of the first Emergency Department Visit: ______________________

2 □ No

- **Hospital Readmissions in the Past 12 Months**

*Were you admitted to the hospital in the past year (excluding day hospital stays)?*

1 □ Yes Date of the first Hospital Readmission: ____________________________

2 □ No
